# Is there still a role for the lung injury score in the era of the Berlin definition ARDS?

**DOI:** 10.1186/2110-5820-4-4

**Published:** 2014-02-18

**Authors:** Kirsten Neudoerffer Kangelaris, Carolyn S Calfee, Addison K May, Hanjing Zhuo, Michael A Matthay, Lorraine B Ware

**Affiliations:** 1 Department of Medicine, Division of Hospital Medicine, University of California, San Francisco 94143-0131, CA, USA; 2 Department of Medicine, Division of Pulmonary and Critical Care, University of California, San Francisco, CA, USA; 3 Department of Anesthesia and Perioperative Care, University of California, San Francisco, CA, USA; 4 Department of Surgery, Vanderbilt University School of Medicine, Nashville, TN, USA; 5 Department of Medicine, Division of Allergy, Pulmonary and Critical Care Medicine, Vanderbilt University School of Medicine, Nashville, TN, USA

**Keywords:** Acute lung injury, Acute respiratory distress syndrome, Lung injury score, Berlin definition, Clinical outcomes, Critical illness

## Abstract

**Background:**

The Lung Injury Score (LIS) remains a commonly utilized measure of lung injury severity though the additive value of LIS to predict ARDS outcomes over the recent Berlin definition of ARDS, which incorporates severity, is not known.

**Methods:**

We tested the association of LIS (in which scores range from 0 to 4, with higher scores indicating more severe lung injury) and its four components calculated on the day of ARDS diagnosis with ARDS morbidity and mortality in a large, multi-ICU cohort of patients with Berlin-defined ARDS. Receiver Operator Characteristic (ROC) curves were generated to compare the predictive validity of LIS for mortality to Berlin stages of severity (mild, moderate and severe).

**Results:**

In 550 ARDS patients, a one-point increase in LIS was associated with 58% increased odds of in-hospital death (95% CI 14 to 219%, *P* = 0.006), a 7% reduction in ventilator-free days (95% CI 2 to 13%, *P* = 0.01), and, among patients surviving hospitalization, a 25% increase in days of mechanical ventilation (95% CI 9 to 43%, *P* = 0.001) and a 16% increase (95% CI 2 to 31%, *P* = 0.02) in the number of ICU days. However, the mean LIS was only 0.2 points higher (95% CI 0.1 to 0.3) among those who died compared to those who lived. Berlin stages of severity were highly correlated with LIS (Spearman’s rho 0.72, *P* < 0.0001) and were also significantly associated with ARDS mortality and similar morbidity measures. The predictive validity of LIS for mortality was similar to Berlin stages of severity with an area under the curve of 0.58 compared to 0.60, respectively (*P*-value 0.49).

**Conclusions:**

In a large, multi-ICU cohort of patients with ARDS, both LIS and the Berlin definition severity stages were associated with increased in-hospital morbidity and mortality. However, predictive validity of both scores was marginal, and there was no additive value of LIS over Berlin. Although neither LIS nor the Berlin definition were designed to prognosticate outcomes, these findings suggest that the role of LIS in characterizing lung injury severity in the era of the Berlin definition ARDS may be limited.

## Background

Mortality in the acute respiratory distress syndrome (ARDS) has declined significantly in the last decade as a result of improved supportive care, treatments for sepsis and multi-organ failure, and the advent of low tidal volume ventilation [1,2]. Accurate clinical measures of severity of ARDS and mortality prediction are necessary to select appropriate patients for clinical trials to detect the beneficial effect of novel therapies [3-6]. Although not designed to prognosticate outcomes, the recent Berlin definition for ARDS was created, in part, to address the need for a consistent measure of severity of ARDS that corresponded with clinical outcomes [7]. Generated using an experimental method combined with a consensus process to define ARDS severity, the Berlin group considered the degree of hypoxemia (PaO_2_/FiO_2_), in combination with ancillary variables for severe ARDS including radiographic severity, respiratory system compliance, positive end-expiratory pressure (PEEP) and corrected expired volume per minute to define lung injury severity. After testing these variables in over 4,000 patients from multiple centers, they found that only hypoxemia contributed to the predictive validity of the definition. These findings raise the question of the utility of these ancillary variables in describing severity of lung injury.

The Lung Injury Score (LIS), proposed in 1988 by Murray and colleagues, [8] has been a commonly utilized measure of acute lung injury (ALI) severity in clinical studies. Derived empirically by expert consensus, the score is composed of four components: 1) chest radiograph; 2) hypoxemia score; 3) PEEP; and 4) static compliance of respiratory system. The LIS preceded the first consensus definition of ALI/ARDS (American-European Consensus Committee (AECC) definition for ALI/ARDS) in 1994, [9] and was designed to measure the pathophysiological features of ARDS; however, it has not been validated as an accurate measure of lung injury severity and its use is not specific to ARDS [10]. Nonetheless, LIS has become a standard measure of ARDS severity that remains widely used. In this capacity, LIS has been presented as a measure of baseline lung injury severity in ARDS clinical studies. In addition, LIS ≥ 3 has been commonly used to identify severe ARDS for consideration of possible rescue therapies, [11-13] and changes in LIS over time have been used as a primary physiologic endpoint to study the efficacy of interventions [12,14]. The original manuscript [8], cited in over 1,400 scholarly articles, has accumulated over 67 new citations since 2012, many of which were published following the announcement of the Berlin definition of ARDS [15].

Although LIS is frequently employed as a measure of the severity of lung injury, its validity in predicting acute lung injury-related outcomes has not been rigorously studied in the era of lung-protective ventilation. Furthermore, the utility of LIS in the context of the recently described Berlin definition of ARDS, [7] which subdivides ARDS into three levels of severity of hypoxemia, is not known. Thus, it is important to determine whether LIS remains a useful measure of lung injury severity in the broad spectrum of patients treated for ARDS in modern clinical practice. This study was designed to compare the association between LIS and Berlin severity stages and in-hospital mortality and morbidity in a large, prospective, multi-ICU cohort of patients meeting the Berlin definition of ARDS.

## Methods

### Subjects

We analyzed data drawn from a multi-ICU, prospective cohort study, entitled the Validation of biomarkers in Acute Lung Injury Diagnosis (VALID) study, the primary purpose of which is to identify biomarkers of diagnosis and prognosis in ARDS. Patients at Vanderbilt University Medical Center (VUMC) were enrolled between January 2006 and February 2011, and details of study enrollment and informed consent have been described previously [16-18]. Briefly, enrolled patients were admitted to the medical, surgical, trauma or cardiovascular ICUs at VUMC if they remained in the ICU the morning of day two. Exclusions included ICU stay greater than 48 hours prior to VUMC ICU admission, uncomplicated overdose, severe chronic lung disease, plans to transfer out of ICU on day two and non-mechanically ventilated or post-surgical patients in the cardiovascular ICU.

**Figure 1:**
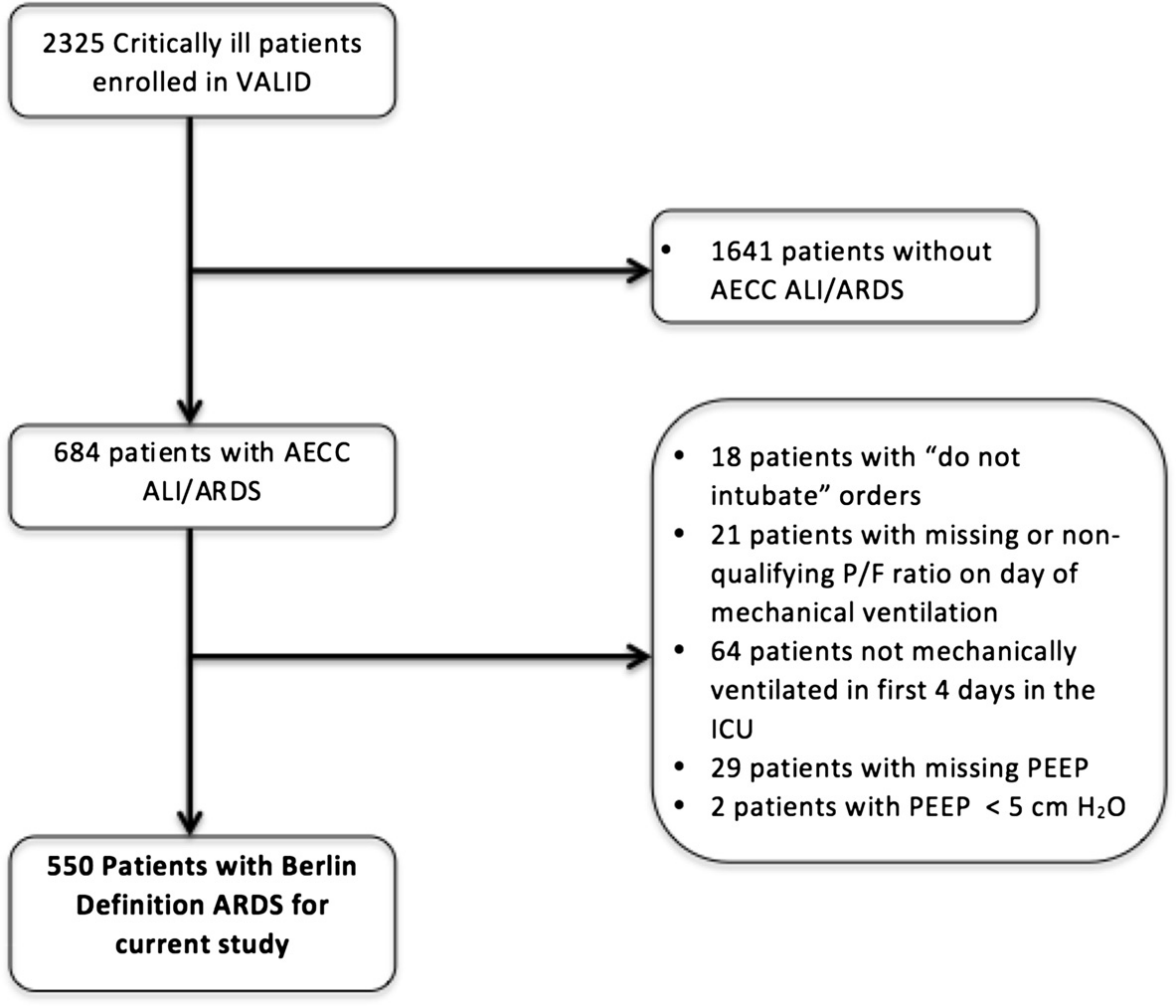
Flow diagram of the patients included in the current study.

For this analysis, we included patients who met the Berlin definition of ARDS [7]. Patients were classified according to Berlin level of severity as mild (PaO_2_/FiO_2_ 200 to ≤ 300 mmHg with PEEP ≥ 5 cm H_2_O or continuous positive airway pressure (CPAP) ≥ 5 cm H_2_O), moderate (PaO_2_/FiO_2_ 100 to ≤ 200 mmHg with PEEP ≥ 5 cm H_2_O), and severe (PaO_2_/FiO_2_ ≤ 100 mmHg with PEEP ≥ 5 cm H_2_O). The diagnosis of ARDS could be established at any time during the first four days in the ICU. For diagnosis, the ratio of pulse oximetric saturation to fraction of inspired oxygen (SpO_2_/FiO_2_) was used as a validated surrogate for PaO_2_/FiO_2_ among patients without an arterial blood gas measurement at the time of ARDS diagnosis: SpO_2_/FiO_2_ = 64 + 0.84 × (PaO_2_/FiO_2_) [19]. Among 2,325 patients enrolled in VALID, we included 550 patients who met Berlin definition criteria for ARDS (Flow diagram Figure 1).

The Institutional Review Board (IRB) at Vanderbilt University (IRB#051065) approved the study.

### Informed consent

Informed consent was obtained from patients or their surrogates whenever possible. For patients who were unable to provide informed consent due to their clinical condition and for whom no surrogates were available, a waiver of informed consent was granted by the IRB due to the minimal risk of the observational study.

### Lung Injury Score

The Lung Injury Score and each of its components [8] and Berlin severity levels were calculated from the most severe measures on the day of ARDS diagnosis or on the initial enrollment day in VALID, whichever came last. For LIS, each of the four components is categorized from 0 to 4, where a higher number is worse (Table 1). The total LIS is obtained by dividing the aggregate sum by the number of components used. For example, if one component is unavailable then the LIS would be the sum of the three other components divided by three.

**Table 1:** **Components of the murray lung injury score **[8]

	**Value**	
1. Chest radiograph score		
No alveolar consolidation		0
Alveolar consolidation confined to 1 quadrant		1
Alveolar consolidation confined to 2 quadrants		2
Alveolar consolidation confined to 3 quadrants		3
Alveolar consolidation in all 4 quadrants		4
2. Hypoxemia score ^a^		
PaO_2_/FIO_2_	≥ 300	0
PaO_2_/FIO_2_	225 to 299	1
PaO_2_/FIO_2_	175 to 224	2
PaO_2_/FIO_2_	100 to 174	3
PaO_2_/FIO_2_	< 100	4
3. PEEP score (when ventilated)		
PEEP	≤ 5 cm H_2_O	0
PEEP	6 to 8 cm H_2_O	1
PEEP	9 to 11 cm H_2_O	2
PEEP	12 to 14 cm H_2_O	3
PEEP	≥ 15 cm H_2_O	4
4. Respiratory system compliance score (when available)		
Compliance	≥ 80 ml/cm H_2_O	0
Compliance	60 to 79 ml/cm H_2_O	1
Compliance	40 to 59 ml/cm H_2_O	2
Compliance	20 to 39 ml/cm H_2_O	3
Compliance	≤ 19 ml/cm H_2_O	4

The final value is obtained by dividing the aggregate sum by the number of components.

Abbreviations: *PaO*_*2*_*/FIO*_*2*_ = arterial oxygen tension to inspired oxygen concentration ratio, *PEEP* = positive end-expiratory pressure.

^a^SpO2/FiO2 equivalent [19] used where qualifying PaO_2_/FIO_2_ not available.

Table adapted from Murray *et al*. Am Rev Respir Disease 138:720–723, erratum 1989:139:1065 [8].

### Outcomes

The primary outcome was in-hospital mortality, defined as death during the incident hospitalization. Secondary outcomes included 28-day ventilator-free days (VFD), defined as the number of days alive and free of mechanical ventilation to day 28, with VFD = 0 for patients who died in the first four weeks, [20] and among survivors to hospital discharge: days of mechanical ventilation; days in ICU; and length of hospital stay.

### Other prognostic indices

We compared LIS and Berlin level of severity to two well-validated, general severity of illness scores: (1) Acute Physiology and Chronic Health Evaluation (APACHE) II, [21] and (2) Simplified Acute Physiology Score (SAPS) II [22]. Both scores were calculated on the day of enrollment into VALID on the morning of ICU day two.

### Definitions of ARDS risk factors

Sepsis was defined according to consensus definition [23] as evidence of infection and at least two criteria of systemic inflammatory response syndrome. Trauma was defined as major blunt or penetrating traumatic injury necessitating admission to the trauma ICU. To diagnose pneumonia, two or more of the following criteria were required: (1) new infiltrate on chest radiograph; (2) temperature higher than 38°C or lower than 36°C or white blood counts more than 12,000/μl, less than 4,000/μl or band forms more than 10%; (3) positive microbiologic culture. Aspiration was defined as witnessed or suspected aspiration events or retrieval of gastric contents from the airway or endotracheal tube.

### Statistical analysis

For all analyses, the LIS score was treated as a continuous variable and LIS components as ordinal variables. Berlin severity levels were treated as ordinal variables. To determine differences in LIS score according to in-hospital mortality, we used the Student’s *t*-test. For LIS components and Berlin severity levels, *P*-values were generated using the Wilcoxon-rank sum test. The association between LIS and secondary outcomes was tested using the Spearman correlation test. We compared the discrimination of LIS and Berlin severity to the general severity scores using Receiver Operating Characteristic (ROC) curves and used the test of equality of ROC areas to determine differences across severity indices. Calibration of the model to evaluate the concordance of observed and predicted mortality was evaluated with Hosmer-Lemeshow goodness-of-fit test, and mortality was categorized according to four different LIS categories (0 to 1.0; 1.1 to 2.0; 2.1 to 3.0; > 3.0) to identify whether there was trend between increasing mortality with each increased point in the LIS score.

As a sensitivity analysis, we tested the prognostic value of LIS in two 'severe ARDS’ subgroups as defined as (1) LIS ≥ 3, a cut-point used to identify 'refractory ARDS’ for consideration of possible rescue therapies; [11-13] and (2) PEEP values ≥ 10 cm H_2_O, a cutoff which has been associated with improved consistency in measurement of hypoxemia [24].

Analyses were performed using STATA version 12.1 (STATA Corp, College Station, TX, USA). Statistical significance was defined as a two-tailed *P* < 0.05 for all analyses.

## Results

**Figure 2:**
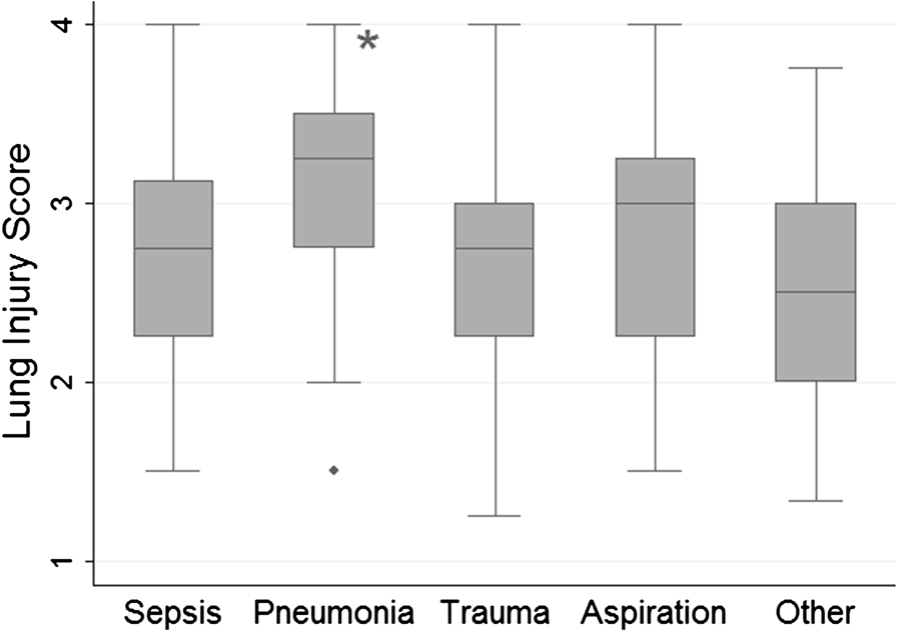
**Box plot comparison of Lung Injury Score according to acute respiratory distress syndrome (ARDS) risk factor in 550 patients with Berlin-defined ARDS.** The line in the middle of the box represents the median and the lines that form the box correspond to the 25th and 75th percentiles. The LIS differs significantly across ARDS risk factor group, which is driven by an increased LIS among patients with pneumonia, *P* < 0.0001. Compared to all other causes of ARDS, mean baseline LIS for patients with pneumonia as a primary cause of ARDS is 0.5 points higher (95% CI 0.3 to 0.6).

Table 2 shows baseline characteristics among 550 patients with ARDS included in this study. The sample was 59% male and 85% white. Trauma, sepsis, and pneumonia were the top ARDS risk factors, and mean LIS differed significantly across ARDS risk factor groups, driven by a higher mean LIS score among patients with pneumonia (*P* < 0.0001) (Figure 2). Patients who died in the hospital were older, more likely to be white (which was accounted for by racial differences in ARDS risk factor), had increased severity of illness on presentation, and were more likely to have sepsis and less likely to have trauma as a risk factor for ARDS (Table 2).

**Table 2:** Baseline characteristics among 550 patients with acute respiratory distress syndrome (ARDS)

**Characteristic**	**Total cohort = 550 (%)**	**Survived to discharge**	**Died in the hospital**	***P*-value**
		**(N = 415)**	**(N = 135)**	
Age, mean ± SD	51 ± 18	49 ± 18	56 ± 17	< 0.001
Male	322 (59%)	247 (60%)	75 (56%)	0.42
White race	469 (85%)	432 (82%)	127 (94%)	0.01
Primary ARDS risk factor^a^				< 0.001
Sepsis	152 (27%)	104 (25%)	48 (36%)	
Pneumonia	98 (18%)	69 (17%)	29 (21%)	
Trauma	189 (34%)	165 (40%)	24 (18%)	
Aspiration	73 (13%)	52 (13%)	21 (16%)	
Other^b^	38 (7%)	25 (6%)	13 (10%)	
Non-invasive ventilation only on day of ARDS diagnosis	4 (1%)	1 (0%)	3 (2%)	0.02
Sepsis within first 72 hours of enrollment	357 (66%)	265 (64%)	97 (72%)	0.09
PaO_2_/FiO_2_ on the day of ARDS diagnosis, mean ± SD^c^	157 ± 81	161 ± 82	145 ± 77	0.07
SpO_2_/FiO_2_ on the day of ARDS diagnosis, mean ± SD^d^	169 ± 62	175 ± 59	151 ± 66	< 0.001
PEEP, mean ± SD	10 ± 4	10 ± 4	11 ± 4	0.003
Static compliance of the respiratory system, mean ± SD^e^	34 ± 28	34 ± 27	34 ± 30	0.95
APACHE II, mean ± SD	29 ± 8	28 ± 7	32 ± 7	< 0.001
SAPS II, mean ± SD	57 ± 16	55 ± 15	63 ± 16	< 0.001

Abbreviations: *APACHE* Acute Physiology and Chronic Health Evaluation, *ARDS* acute respiratory distress syndrome, *IQR* interquartile range, *PaO*_*2*_*/FiO*_*2*,_ partial pressure of arterial oxygen to the fraction of inspired oxygen, *PEEP* positive end-expiratory pressure, *SAPS* Simplified Acute Physiology Score, *SD* standard deviation.

Presented as N (%) unless otherwise specified.

^a^Percentages not adding to 100 are due to rounding.

^b^Includes pancreatitis, near drowning, transfusion-related, and so on.

^c^ PaO_2_/FiO_2_ among 469 patients.

^d^SpO_2_/FiO_2_ among 512 patients.

^e^Among 515 patients.

### LIS and clinical outcomes

The mean LIS was 2.7 ± 0.6 in hospital survivors (N = 415) compared to 2.9 ± 0.6 in non-survivors (N = 135) (Table 3). This 0.2 point difference (95% CI 0.1 to 0.3) was statistically significant *P* = 0.006. The association between LIS and in-hospital death was not modified by the ARDS risk factor (*P* = 0.19 for heterogeneity) or the presence of sepsis in the first four days after enrollment (*P* = 0.68). The LIS components most strongly associated with overall mortality were the PaO_2_/FiO_2_ (*P* < 0.001) and the level of PEEP (*P* = 0.02). The chest radiograph and compliance scores did not differ according to mortality (Table 3). A one-point increase in LIS was associated with a 58% increased odds of in-hospital death (OR 1.58, 95% CI 1.14 to 2.19, *P* = 0.006).

**Table 3:** **Lung Injury Score (LIS) and component scores according to in-hospital mortality in 550 patients on day of acute respiratory distress syndrome (ARDS) diagnosis**^**a**^

**Overall**	**Died N = 135**	**Lived N = 415**	***P*-value**
LIS score, mean ± SD	2.9 ± 0.6	2.7 ± 0.6	0.006^b^
Chest radiograph score	4 (3 to 4)	4 (3 to 4)	0.77
PaO_2_/FiO_2_ category	4 (3 to 4)	3 (2 to 4)	< 0.001
PEEP category	2 (1 to 3)	2 (0 to 3)	0.02
Compliance category	3 (3 to 3)	3 (3 to 3)	0.48

^a^Presented as median (interquartile range) and *P*-value presented as Wilcoxon-rank sum unless otherwise specified. *P*-values are similar using the Student’s *t*-test.

^b^*P*-value generated using the Student’s *t*-test. *P* = 0.004 using Wilcoxon-rank sum statistic.

Among secondary outcomes evaluated, VFDs were inversely associated with LIS (Spearman’s rho = -0.17, *P* = 0.0001) with a 7% reduction in VFD (95% CI 2 to 13%, *P*-value = 0.01) for every one-point increase in LIS. Among 415 hospital survivors, the LIS was positively associated with days of mechanical ventilation (Spearman’s rho = 0.17, *P* = 0.0004) and days in the ICU (Spearman’s rho = 0.13, *P* = 0.007). For every one-point increase in LIS, there was a 25% increase (95% CI 9 to 43%, *P* = 0.001) in number of days of mechanical ventilation and a 16% increase (95% CI 2 to 31%, *P* = 0.02) in number of ICU days. The LIS did not predict total days of hospitalization (Spearman’s rho = 0.07, *P* = 0.15) among hospital survivors.

### LIS and Berlin definition

**Figure 3:**
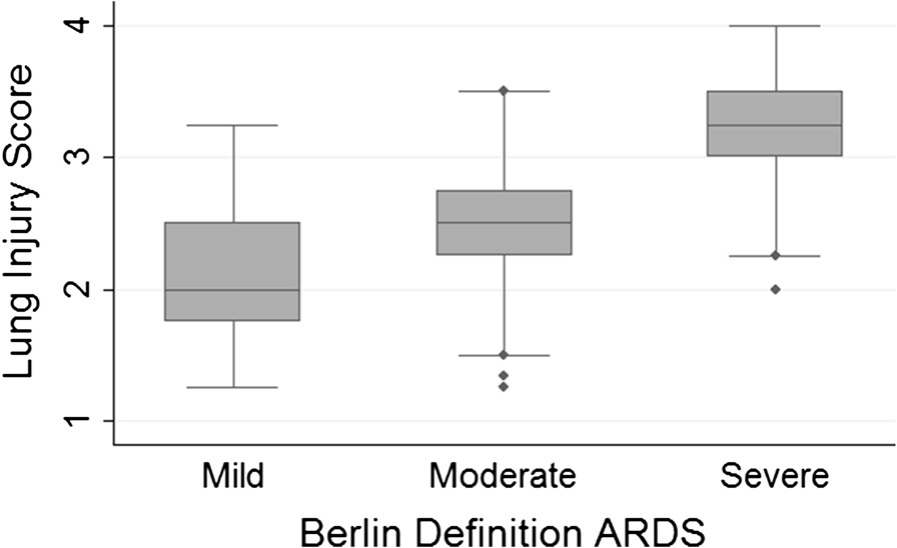
**Box plot comparison of Lung Injury Score according to Berlin definition severity in 550 patients with Berlin definition acute respiratory distress syndrome (ARDS).** The line in the middle of the box represents the median and the lines that form the box correspond to the 25th and 75th percentiles. The LIS increases with increase Berlin definition severity, *P* < 0.001 for trend.

LIS was highly correlated with the Berlin oxygenation criteria (Spearman’s rho of 0.72, *P* < 0.0001). LIS increased with each increased level of Berlin severity (Figure 3 and Table 4). Among patients characterized by the Berlin definition as mild, the mean LIS was 2.1 ± 0.4 compared to a mean LIS of 2.5 ± 0.5 in moderate and 3.3 ± 0.4 in severe Berlin definition ARDS (*P* < 0.001 for trend).

**Table 4:** Predictive validity of Berlin definition acute respiratory distress syndrome (ARDS) patients in Validation of biomarkers in Acute Lung Injury Diagnosis (VALID)

	**Mild**	**Moderate**	**Severe**	***P*-value**^**a**^
Overall number (%) of patients, N = 550	76 (14)	257 (47)	217 (39)	
Lung Injury Score, mean ± SD	2.1 ± 0.4	2.5 ± 0.5	3.3 ± 0.4	< 0.001
Mortality, number (%)	14 (18)	48 (19)	73 (34)	< 0.001
Ventilator-free days, median (IQR)	20 (7.5 to 25)	18 (9 to 23)	14 (0 to 22)	< 0.001
Number (%) of survivors, N = 415	62 (15)	209 (50)	144 (35)	
Duration of mechanical ventilation in survivors, median (IQR)	6 (3 to 12)	7 (4 to 13)	8 (5 to 14.5)	0.006
ICU days in survivors, median (IQR)	9 (6 to 15)	10 (6 to 16)	11.5 (7 to 19)	0.07
Length of hospitalization in survivors, median (IQR)	16 (10 to 23)	16 (11 to 26)	19 (13 to 29)	0.08

^a^Test of trend.

Abbreviations: *ARDS* = acute respiratory distress syndrome, *ICU* = intensive care unit, *IQR* = interquartile range, *SD* = standard deviation.

An increased Berlin level of ARDS severity was associated with worse clinical outcomes in ARDS including increased in-hospital mortality, decreased ventilator-free days, and increased duration of mechanical ventilation in survivors (Table 4).

### Comparison of LIS and Berlin severity to general severity scores

**Figure 4:**
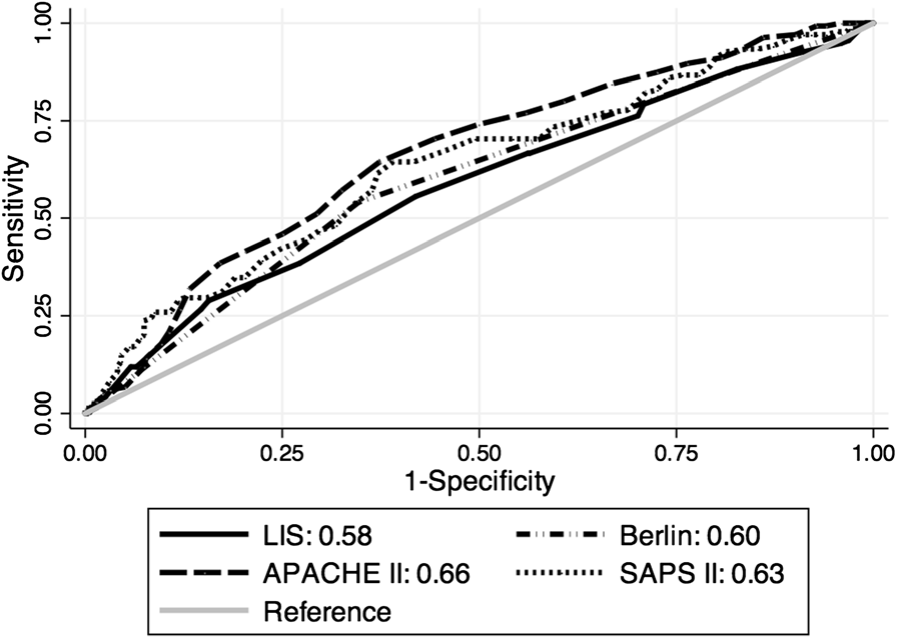
**Comparison of Receiver Operating Characteristic Curves of Lung Injury Score (LIS) to Berlin severity, APACHE II and SAPS II.** Discrimination of LIS for mortality is similar to Berlin severity (*P* = 0.47) and SAPS II (*P* = 0.22) and inferior to APACHE II (*P* = 0.04).

**Figure 5:**
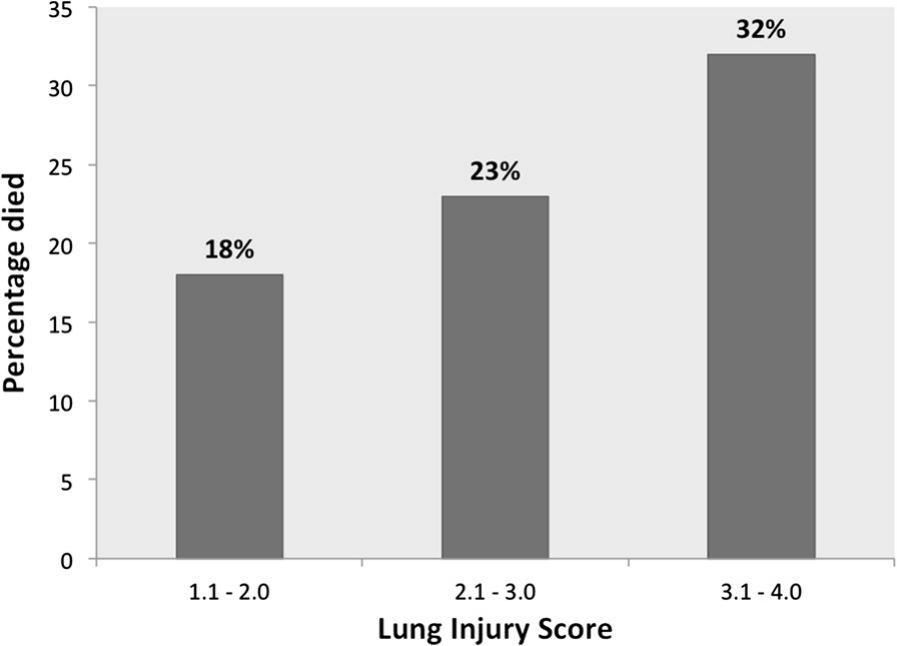
**Categories of Lung Injury Score (LIS) by in-hospital mortality in 550 patients with acute respiratory distress syndrome (ARDS).** No patients with LIS between 0 and 1.

The area under the ROC curve (AUC) of LIS for hospital mortality was 0.58 (95% CI 0.53 to 0.64) compared to an AUC of 0.60 (95% CI 0.55 to 0.65) for the Berlin severity scores. There was no statistically significant difference between these values, *P*-value = 0.47. Compared to the LIS, the AUC for APACHE II 0.66 (0.61 to 0.71) was significantly higher (*P* = 0.04); the AUC for SAPS II was also higher (0.63, 95% CI 0.58 to 0.69) but this difference was not statistically significant (*P* = 0.22) (Figure 4). Calibration was adequate for LIS, Berlin severity and both severity of illness scores, with similar expected to observed mortality across subgroups (data not shown). All scores passed the Hosmer-Lemeshow goodness-of-fit test with *P*-values > 0.25. Figure 5 demonstrates that mortality increased for each one-point increase in LIS.

### Sensitivity analyses

Using a cut-point of a LIS score of ≥ 3, high LIS was present in 45% (N = 249) of patients on the day of ARDS diagnosis, with 30% mortality in the high LIS group compared with 20% mortality in the lower LIS group (*P* = 0.006). The AUC at this cut-point was 0.57 (95% CI 0.52 to 0.62). Discrimination of LIS was not improved in 342 (62%) patients with PEEP values ≥ 10 cm H_2_O (AUC 0.58, 95% CI 0.51 to 0.65).

## Discussion

Although the four-point LIS was never intended as a prognostic tool, it has been used as a measure of the severity of lung injury that thereby infers prognostic value. The recently developed Berlin definition of ARDS includes a measure of lung injury severity based on three levels of hypoxemia and baseline PEEP of at least 5 cm H_2_O [7]. Our study tested the predictive validity of LIS on mortality and morbidity in a large, heterogeneous group of patients with Berlin definition ARDS. We found that although LIS at the time of ARDS diagnosis was associated with in-hospital mortality, the difference between mean LIS in those who died and lived was only 0.2 points, a difference of minimal clinical significance. Of the four LIS components, only PaO_2_/FiO_2_ and PEEP categories were associated with mortality overall. The discrimination of LIS for in-hospital mortality was comparable to the Berlin severity scale and only marginally better than chance alone. The predictive validity was not improved when evaluated in more severe subgroups such as those with higher PEEP or LIS ≥ 3. We also found that LIS was predictive of the duration of mechanical ventilation and days in the ICU. This finding was important, as LIS may be more suited to discriminate pulmonary-specific outcomes. The Berlin definition severity stages were associated with mortality and also found to be associated with increased mechanical ventilation requirements. The predictive validity of Berlin criteria was similarly marginal and failed to identify three distinct mortality classes with a mortality of 18% in mild versus 19% in moderate ARDS.

Overall, our findings are consistent with the findings presented in the development of the Berlin definition for ARDS [7]. Although several components included in the LIS were considered for the Berlin definition, including severity of radiographic criteria, higher levels of PEEP, and static respiratory compliance, they were ultimately dropped in the final Berlin definition for lack of additive predictive value. Similarly, we found that only PEEP category and level of hypoxemia in the LIS were associated with mortality. We also found that both LIS and the Berlin definition were associated with the duration of mechanical ventilation. Only LIS was significantly associated with duration of ICU stay among survivors, though this correlation was weak and there was a similar, non-statistical trend for the Berlin severity stages.

Clinical measures of severity of lung injury have limitations. First, measures of lung injury will not perform well as prognostic measures because non-pulmonary factors including age, severity of sepsis, co-morbidities and non-pulmonary organ failure remain the most influential predictors of hospital mortality in ARDS, [25-39] and non-resolving respiratory failure accounts for less than 20% of ARDS deaths [34-36]. Also, although the finding that PaO_2_/FiO_2_ level is associated with mortality is consistent with the findings reported from cohorts used for empirical analysis in the development of the Berlin ARDS definition, [40-46] this has not been demonstrated consistently, a finding that may be attributable, in part, to practice variability in mechanical ventilation settings, which is known to have a large effect on PaO_2_/FiO_2_ levels [5,6,24,47,48]. Furthermore, post-mortem studies highlight the poor accuracy that clinical definitions such as the Berlin criteria have for histological definitions of diffuse alveolar damage, which are found in only a minority of patients with clinical ARDS [49,50]. Nonetheless, clinical measures of lung injury severity remain necessary to identify patients for ARDS treatments and clinical trials.

To our knowledge, our study is the first to report a statistically significant association between LIS and mortality in ARDS [5,24,26,28,31,41,51]. Prior studies were limited by small sample size (largest N = 259), mostly considered only a subset of patients with ARDS (PaO_2_/FIO_2_ < 200 mmHg,) [26,28,31,51], and were largely conducted prior to recent treatment advancements including low tidal volume ventilation. At least two studies demonstrated a similarly increased LIS in non-survivors of ARDS compared to survivors as observed in the current study (approximately 0.2 points), but did not have the power to detect a statistically significant difference [5,41]. Furthermore, a study by Villar *et al*. identified LIS as an independent predictor of developing established AECC-defined ARDS after one day of standardized ventilator management [24], which is consistent with our finding that LIS predicts pulmonary outcomes. The lack of a significant association between LIS and clinical outcomes in these smaller studies underscores our finding that LIS may be a marginal though not clinically relevant predictor of outcomes. Finally, it is also possible that we detected an association between LIS and clinical outcomes as a result of limiting our sample to patients meeting the Berlin definition of ARDS, a definition that was created to improve the validity and reliability of the ARDS diagnosis [7].

It is important to note that the mortality rate in our study was lower than in several other epidemiological studies on ARDS [7,52-55]. This was in part due to the high proportion of patients with trauma-related ARDS, for whom the in-hospital mortality rate was 13%. Also, our outcome was in-hospital mortality rather than 60- or 90-day mortality in some other studies. However, even with a lower overall mortality, we detected a statistically significant association between both LIS and Berlin severity and both in-hospital death and more prolonged respiratory failure, demonstrating sufficient power. We recently demonstrated that death after discharge from ARDS hospitalization is more related to underlying co-morbid illness and age rather than severity of ARDS and determined that in-hospital mortality would be more sensitive to measures of lung injury severity [56]. Furthermore, using an endpoint of mortality at 90 days did not change our results (data not shown) although overall mortality increased by 10%.

Despite a statistically significant association between LIS and outcomes, these results should be considered in view of the current use of LIS for clinical and research applications in the context of a the new Berlin definition for ARDS. A single LIS measurement on initial diagnosis of ARDS did not provide additive prognostic information over the three severity categories of the Berlin definition alone. In addition, with only marginal discrimination for mortality, our results do not clearly support the use of LIS cut-points used to define refractory ARDS for consideration of experimental approaches such as extracorporeal membrane oxygenation [13].

Several limitations of the study deserve mention. First, it was performed at a single, tertiary care site; therefore, generalizing the results to other settings may be limited. However, using a large, multi-ICU sample, including a broad range of patients with ARDS, is likely to improve generalizability overall. Furthermore, our test of the prognostic value of the Berlin definition severity stages replicated those described in the initial Berlin definition, further suggesting our sample is representative of a broader ARDS population. Second, we did not evaluate the prognostic value of change in LIS over time. Further studies will be required to determine whether measurement of change in LIS is a more useful prognostic indicator as has been previously suggested [57]. Third, our primary outcome was all-cause in-hospital mortality, so we were not able to directly assess the prognostic value of LIS in identifying the minority of patients with ARDS who die of respiratory failure. However, secondary outcomes of VFD and days of mechanical ventilation were assessed as measures of pulmonary-specific outcomes. Lastly, our general severity scores were generated over the first 24 hours of enrollment, whereas LIS was calculated on the day of ARDS diagnosis. This was the same day for the vast majority (N = 419, 76%) of the study cohort. However, the time difference for the 24% should provide an advantage for LIS as a prognostic marker. Therefore, improved test characteristics in the general severity scores compared to LIS may have been an underestimate of this difference.

## Conclusions

In conclusion, the LIS remains a widely utilized measure of initial lung injury severity in ARDS but does not provide additional prognostic value for mortality or duration of mechanical ventilation compared to the Berlin definition of ARDS. White the four-point LIS may still have value for research purposes to more completely define abnormal lung physiology, it has limited value for estimating prognosis in ARDS patients.

## Abbreviations

AECC: American-European Consensus Committee; ALI: acute lung injury; ARDS: acute respiratory distress syndrome; APACHE: Acute Physiology and Chronic Health Evaluation; AUC: area under the curve; CPAP: continuous positive airway pressure; IRB: Institutional Review Board; LIS: Lung Injury Score; PaO2/FiO2: ratio of arterial oxygen concentration to the fraction of inspired oxygen; PEEP: positive end-expiratory pressure; ROC: Receiver Operator Characteristic; SAPS: Simplified Acute Physiology Score; SpO2/FiO2: ratio of the pulse oximetric saturation to the fraction of inspired oxygen; VALID: Validation of biomarkers in Acute Lung Injury Diagnosis; VFD: ventilator-free days; VUMC: Vanderbilt University Medical Center.

## Competing interests

No author (KNK, AM, HZ, MAM, LBW) reports a conflict of interest. Dr. Calfee has served on medical advisory boards for Cerus Corp and Glaxo Smith Kline.

## Authors’ contributions

KNK, CSC, MAM and LBW contributed to study design, data analysis and interpretation and drafting and revising the manuscript critically for important intellectual content. HZ and AKM contributed to acquisition of data, and analysis and interpretation of data and revising the manuscript. All authors approved the manuscript to be published, and KNK is the guarantor of the entire manuscript.
